# Smallest *Anopheles farauti* occur during the peak transmission season in the Solomon Islands

**DOI:** 10.1186/s12936-019-2847-2

**Published:** 2019-06-24

**Authors:** Kimberley McLaughlin, Tanya L. Russell, Allan Apairamo, Hugo Bugoro, Jance Oscar, Robert D. Cooper, Nigel W. Beebe, Scott A. Ritchie, Thomas R. Burkot

**Affiliations:** 1 Australian Institute of Tropical Health and Medicine, James Cook University, Cairns, QLD 4870 Australia; 2 National Vector Borne Disease Control Programme, Ministry of Health and Medical Services, Honiara, Solomon Islands; 3 Research Department, Solomon Islands National University, Honiara, Solomon Islands; 4 Western Province Malaria Control, Gizo, Western Province, Solomon Islands; 5 Australian Defense Force Malaria and Infectious Disease Institute, Gallipoli Barracks, Enoggera, 4052 Australia; 6 School of Biological Sciences, University of Queensland, St. Lucia, QLD 4068 Australia; 7 CSIRO, Dutton Park, Brisbane, QLD 4001 Australia

**Keywords:** *Anopheles farauti*, *Anopheles hinesorum*, *Anopheles lungae*, Density-dependence, Wing length, Size variation, Solomon Islands

## Abstract

**Background:**

Malaria transmission varies in intensity amongst Solomon Island villages where *Anopheles farauti* is the only vector. This variation in transmission intensity might be explained by density-dependent processes during *An. farauti* larval development, as density dependence can impact adult size with associated fitness costs and daily survivorship.

**Methods:**

Adult anophelines were sampled from six villages in Western and Central Provinces, Solomon Islands between March 2014 and February 2017. The size of females was estimated by measuring wing lengths, and then analysed for associations with biting densities and rainfall.

**Results:**

In the Solomon Islands, three anopheline species, *An. farauti*, *Anopheles hinesorum* and *Anopheles lungae*, differed in size. The primary malaria vector, *An. farauti*, varied significantly in size among villages. Greater rainfall was directly associated with higher densities of *An. farauti* biting rates, but inversely associated with body size with the smallest mean sized mosquitoes present during the peak transmission period. A measurable association between body size and survivorship was not found.

**Conclusions:**

Density dependent effects are likely impacting the size of adult *An. farauti* emerging from a range of larval habitats. The data suggest that rainfall increases *An. farauti* numbers and that these more abundant mosquitoes are significantly smaller in size, but without any reduced survivorship being associated with smaller size. The higher malaria transmission rate in a high malaria focus village appears to be determined more by vector numbers than size or survivorship of the vectors.

**Electronic supplementary material:**

The online version of this article (10.1186/s12936-019-2847-2) contains supplementary material, which is available to authorized users.

## Background

Vector control with indoor residual spraying (IRS) and insecticide-treated nets (ITNs) is responsible for 80% of the reduction in *Plasmodium falciparum* cases in Africa between 2000 and 2015 [1]. The global malaria cases have since stabilized. Further reductions in malaria cases will require strengthened malaria control [2, 3]. ITNs are most effective against vectors that blood feed indoors and late at night, while IRS is most effective when vectors rest indoors [3]. Despite increasing prevalence of insecticide resistance (physiological and behavioural), LLINs and IRS remain sufficiently effective to provide significant malaria control [4–6]. At the present time, the only WHO-recommended strategy that targets malaria vectors outside of houses is larval source management (LSM); but LSM is only recommended in areas with seasonal transmission or where the larval habitats are few in number, fixed in location and easily accessible (including urban areas) [7]. Because LSM is difficult to implement effectively in many environments, it is only recommended as a supplement to LLINs or IRS [3].

Maintaining effective vector control will require understanding vector ecologies and behaviours to select interventions that target vulnerabilities in the vectors’ behaviours [8, 9]. Anopheline populations are strongly influenced by environmental factors (e.g., temperature, water and resource availability) [10–12], and there is growing evidence that anopheline populations are also influenced by density-dependent processes [13–15]. The fitness of adult anopheline mosquitoes (adult survival and fecundity) can be influenced by interacting environmental and density-dependent factors, with fitness directly associated with adult body size [16–18]. The size of adult mosquitoes is governed by competition during the immature aquatic stages; with the body size of emerging adults being directly associated with larval densities (e.g., increased competition at high larval densities leads to the emergence of smaller adult mosquitoes). Smaller adults can be less successful in mating, have reduced fecundity [16, 19, 20] and lower survival rates [21–23] and, consequently, have a lower potential for transmitting malaria [15, 24].

*Anopheles farauti* is the main malaria vector in the Solomon Islands where, despite reductions in transmission, there were 86,000 estimated cases in 2016 [25]. This species oviposits in a wide range of habitats ranging from small ground pools and ditches to large freshwater and brackish swamps [26, 27]. It is hypothesized that density dependence in *An. farauti* larval habitats would be expressed as variations in *An. farauti* adult mosquito size, with density dependence theory predicting larger and more fit mosquitoes being associated with both lower densities in the larval environment and with higher potential for malaria transmission as adults [15, 24, 28].

## Methods

### Study sites

**Fig. 1:**
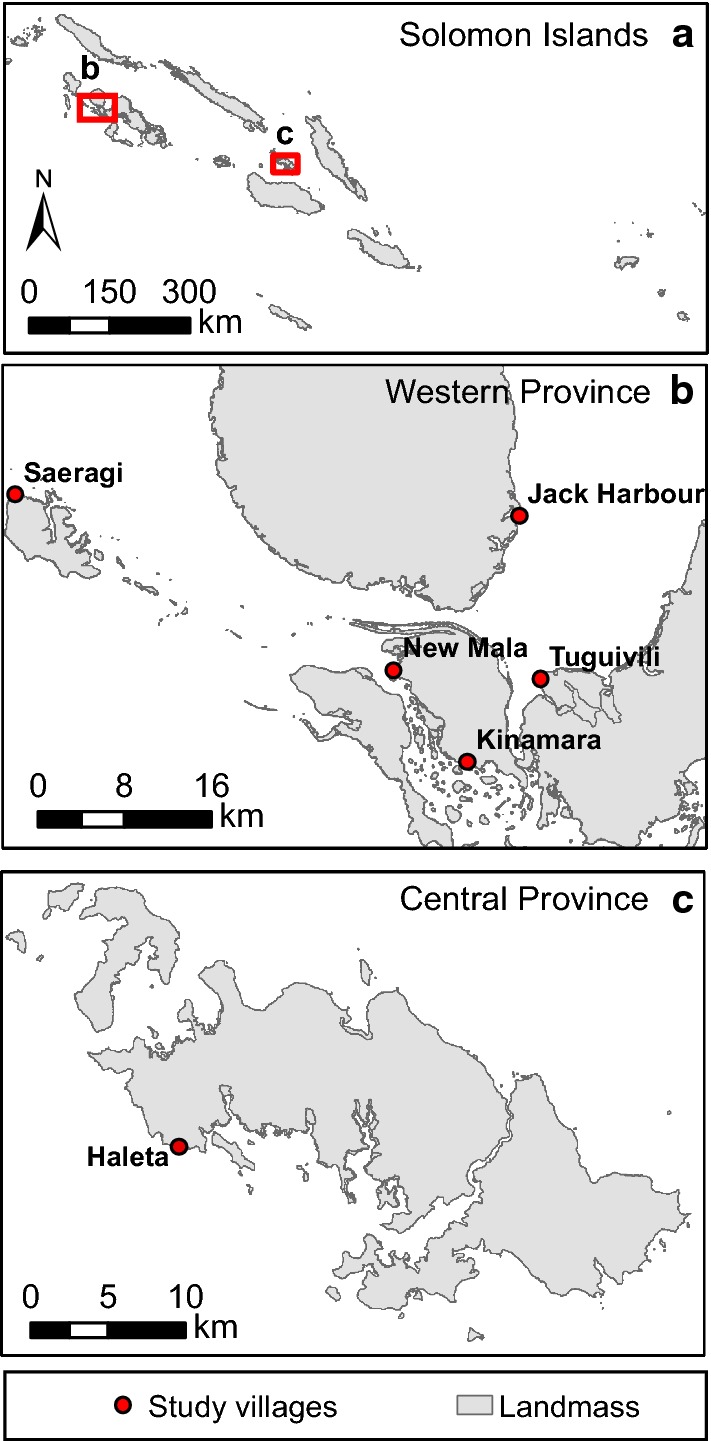
Map of **a** the Solomon Islands showing (**b**) the five study villages in Western Province (8°0′S, 157°0′E) and **c** Haleta village in Central Province (9°0′S, 159°45′E)

The study was conducted in Jack Harbour, Kinamara, New Mala, Saeragi and Tuguivili villages in Western Province (8°0′S, 157°0′E; [29]) and Haleta village in Central Province (9°0′S, 159°45′E; [4]) (Fig. 1). The main malaria vector is *An. farauti*. *Anopheles hinesorum*, *Anopheles lungae* and *Anopheles solomonis* are predominantly zoophagic and not believed to contribute significantly to malaria transmission [27, 29].

The villages are on volcanic, mountainous and rain-forested islands. The climate of the region is hot and wet with an annual rainfall of 3725 mm for New Georgia Island in Western Province and 2837 mm in Central Province (average from 1999 to 2010; Bureau of Meteorology, Solomon Islands, for Munda Airport, Western Province, and Henderson Airport to represent Central Province, unpublished data). The mean daily minimum and maximum temperatures of both provinces were 24 °C and 30 °C, respectively, with an overall mean of 26 °C.

In Western Province the annual parasite incidence (API) was 8 per 1000 population in 2014, increasing to 20 per 1000 population by 2017 (Solomon Islands Ministry of Health and Medical Services (SIMHMS), unpublished data). In this low transmission province, malaria foci have emerged including Jack Harbour village [29]. In Central Province the API was 72 per 1000 population in 2014 increasing to 220 per 100 population by 2017 (SIMHMS, unpublished data). Self-reported LLIN usage was 68% in Western Province (unpublished data) and 73% in Central Province [30].

### Study period

Anophelines were sampled on multiple occasions in each village between July 2015 and July 2017 (Table 1). Adult anopheline densities up to August 2016 were previously reported from these study villages [4, 29]; subsequent collections to July 2017 are updated here.

**Table 1:** Timeline of anopheline surveys in Western and Central Provinces, Solomon Islands

Village	Sample period							
	2015		2016				2017	
	Aug	Nov	Jan	Jun	Aug	Nov	Feb	Jul
Jack Harbour	×				×	×	×	
Kinamara	×	×		×				
New Mala	×			×	×			×
Saeragi	×				×	×		×
Tuguivili		×		×	×	×	×	×
Haleta			×		×			×

### Sampling and processing of adult anophelines

Anophelines were caught using human landing catches (HLC) from 18.00 to 00.00 at 10 outdoor sites that were used during each of the four nights per village during each survey, as previously described [4, 29]. Captured anophelines were identified morphologically [31] before storage in 100% ethanol by collection hour and sample site.

Mosquito size was estimated by measuring wing lengths [16]. Individual specimens were dried on a triple vented petri dish for 5 min. Wings were then mounted on double sided sticky tape on a microscope slide. Using a Nikon SMZ-745T microscope with a scaled eye piece, wings were measured under 6.7× from the alular notch to apical margin (excluding the fringe) along the R1 vein.

Individual mosquitoes were identified to species by PCR using the internal transcribed spacer region II of ribosomal DNA (ITS2) [32]. For villages where only *An. farauti* sensu stricto was captured by HLC, a subset of samples was analysed to confirm species identifications. For villages with more than one anopheline species, all samples for which wing lengths were measured were identified by PCR. The rainfall data was sourced from the Munda Airstrip in Western Province (Bureau of Meteorology, Solomon Islands, unpublished data).

### Statistical analysis

Data on mosquito surveys, wing lengths and molecular analyses are available from the James Cook University Tropical Data Hub [33]. Differences in the species composition between the study villages were compared using a Chi-squared contingency table. Generalized linear models (GLM) with a gaussian distribution were used for the following analyses: (a) differences in wing lengths between mosquito species, and (b) differences in the wing length of *An. farauti* between villages. A GLM with a negative binomial distribution was used to analyse differences in adult biting density between villages. The GLMs and sequential post hoc analyses, Tukey–Kramer HSD, were conducted in SAS JMP V14.0.0.

The relationship between wing lengths and concurrent adult biting densities were directly compared with a Spearman’s rank correlation in villages where the wing lengths of > 200 wings were measured (e.g., Jack Harbour village, Tuguivili and Haleta). Both factors were log(*x *+ 1) transformed prior to analyses. A generalized estimating equation (GEE) compared the relationship between: (a) rainfall and density, and (b) density and wing lengths. Rainfall was summed for the 14-day window prior to the date of mosquito collection. The GEE was conducted using SPSS V24, had a normal distribution and incorporated study period as a random factor.

The mean wing lengths of female anopheline mosquitoes captured at each sampling station were projected geographically in QGIS (v3.4). The spatial analysis was only conducted in Jack Harbour where high densities of *An. farauti* were captured. Clusters of sampling sites where larger mosquitoes were captured were detected using SaTScan (v9.6) using a normal model.

## Results

### Species distributions

**Fig. 2:**
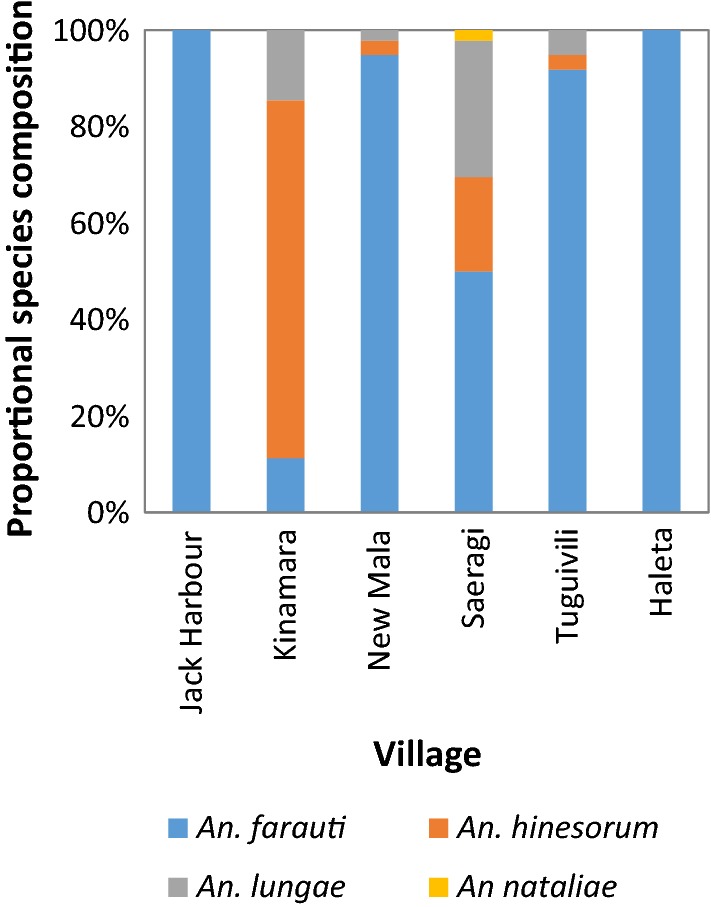
Species compositions of *Anopheles farauti*, *An. hinesorum*, *An. lungae* and *An. nataliae* from Jack Harbour (*n* = 415), Kinamara (*n* = 62), New Mala (*n* = 137), Saeragi (*n* = 46) and Tuguivili (*n* = 294) in Western Province and Haleta (*n* = 89) in Central Province

A total of 10,973 anophelines were collected during 1005 man-nights of HLC collections. Members of both the *An. farauti* sensu lato (s.l.) (*n *= 8529) and *An. lungae* (s.l.) (*n *= 48) complexes were captured in Western Province, while only members of the *An. farauti* complex (*n *= 2396) were captured in Haleta, Central Province. PCR analysis of all members of the *An. farauti* complex confirmed that 93% of specimens were *An. farauti* (*n *= 937/1005) and 7% were *An. hinesorum* (*n *= 68/1005). Of the *An. lungae* complex specimens, 98% were confirmed by PCR as being *An. lungae* (*n *= 40/41) and 2% were *Anopheles nataliae* (*n *= 1/41). Species compositions varied significantly by village (χ^2^ = 5.53, *DF* = 5, *P* < 0.001) (Fig. 2). *Anopheles farauti* was the dominant species in Jack Harbour, Haleta, Tuguivili and New Mala, with 100% of anophelines captured in Jack Harbour and Haleta being *An. farauti*. The dominant species in Kinamara was *An. hinesorum.* In Saeragi there was a mixture of species comprising 50% *An. farauti*, 20% *An. hinesorum*, 28% *An. lungae* and 2% *An. nataliae* (Fig. 2).

### Anopheline species size

**Fig. 3:**
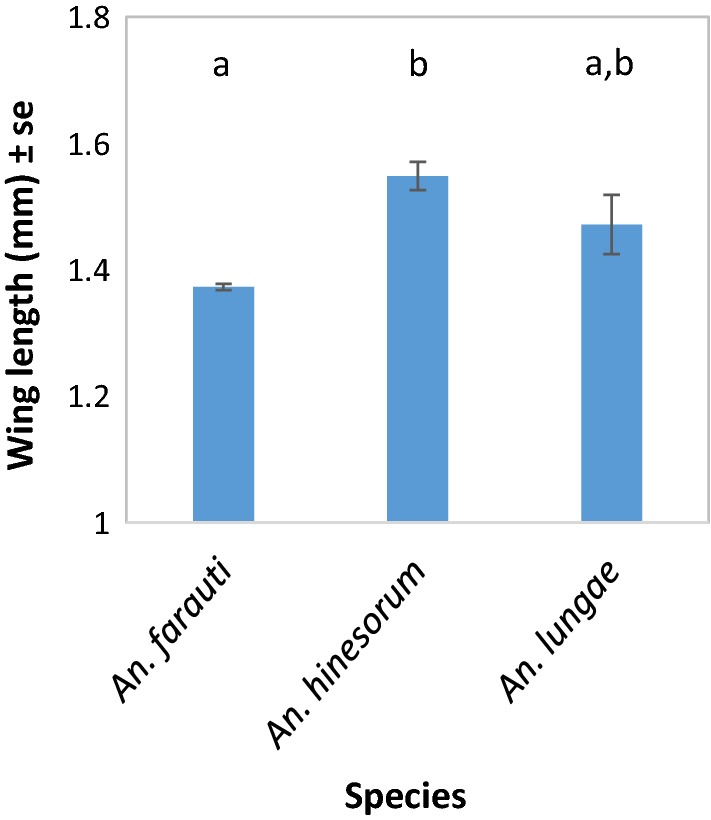
Variation in wing length of *Anopheles farauti* (*n *= 1996), *An. hinesorum* (*n* = 59) and *An. lungae* (*n* = 20) adults. Averages (± se) were calculated across all study villages from Western and Central Provinces, Solomon Islands. Different letters indicate significant differences (*P* < 0.05, Tukey–Kramer HSD) between the wing lengths of the *Anopheles* species

**Fig. 4:**
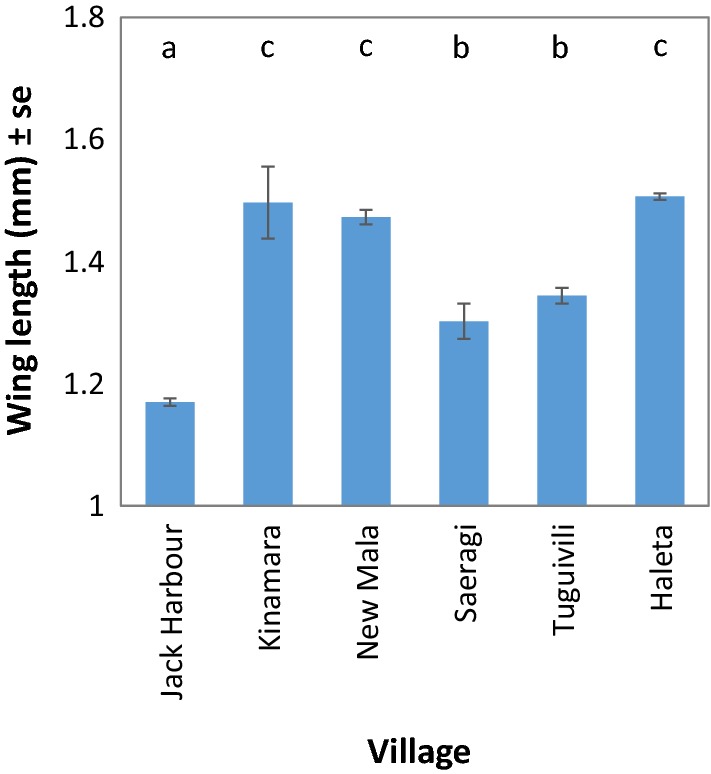
The average wing length (± se) of *Anopheles farauti* specimens sampled from the study villages in Western and Central Provinces, Solomon Islands. Different letters indicate significant differences (*P* < 0.05, Tukey–Kramer HSD) between the wing lengths *An. farauti* captured in different villages

The wings of 2074 female anophelines were measured. Wing length varied significantly by anopheline species (*β* = 0.193, *SE* = 0.0174, *P* < 0.000; Fig. 3) with *An. hinesorum* being significantly larger than *An. farauti* (post hoc: *P* < 0.0001). Mean *An. farauti* size (as determined by wing length) varied by village (*β* = 0.053, *SE* = 0.014, *P* < 0.001; Fig. 4), with the smallest *An. farauti* found in Jack Harbour, while larger *An. farauti* were found in New Mala, Haleta and Kinamara (post hoc: *P *< 0.05).

### Associations of *Anopheles farauti* size with population density, rainfall and distribution

**Fig. 5:**
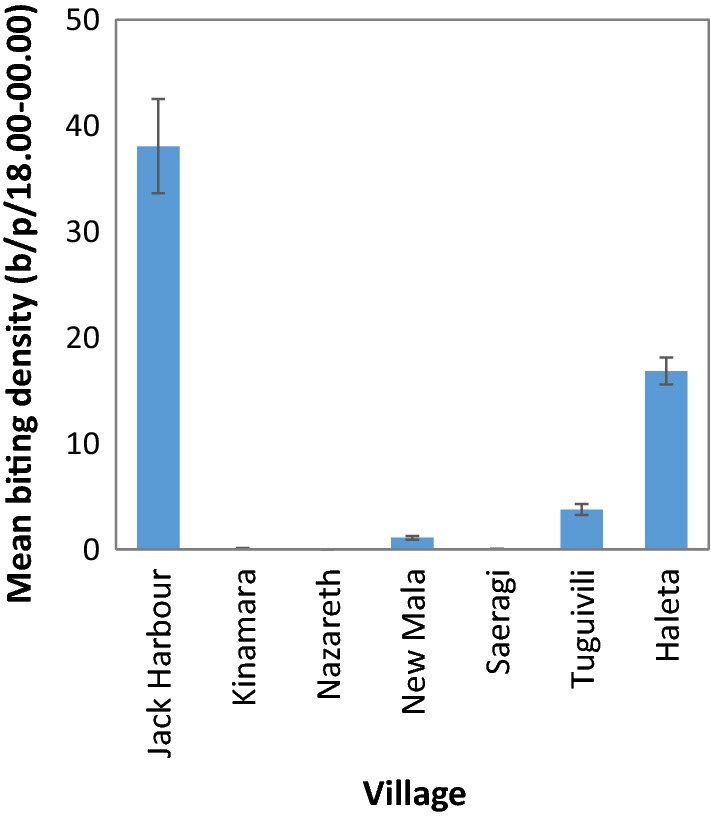
Average (± se) biting density of *Anopheles farauti* in the study villages of Western and Central Province, estimated by human landing catches from 18.00 to 00.00 h

**Fig. 6:**
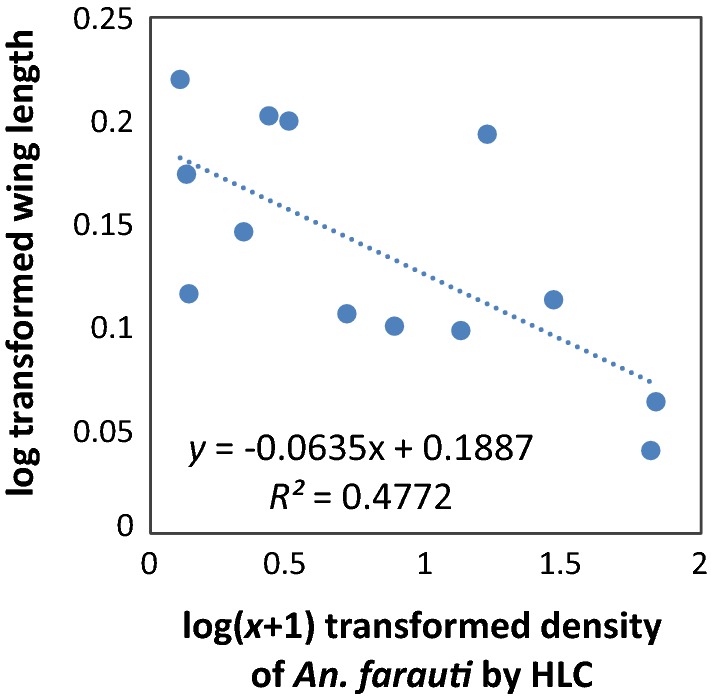
Wing length size and associations with the density of *An. farauti* from Jack Harbour, Tuguivili and Haleta villages as determined by human landing catch

The density of *An. farauti* varied significantly by village (*β* = 1.404, *SE* = 0.5956, *P* < 0.0001; Fig. 5). Highest adult biting densities were found in Jack Harbour, Haleta and Tuguivili. There was a negative exponential correlation between wing length and adult biting density by villages (Fig. 6). After log + 1 transformation, the relationship was linear and the variables were significantly correlated (*r* = − 0.709, *P* = 0.0088).

**Fig. 7:**
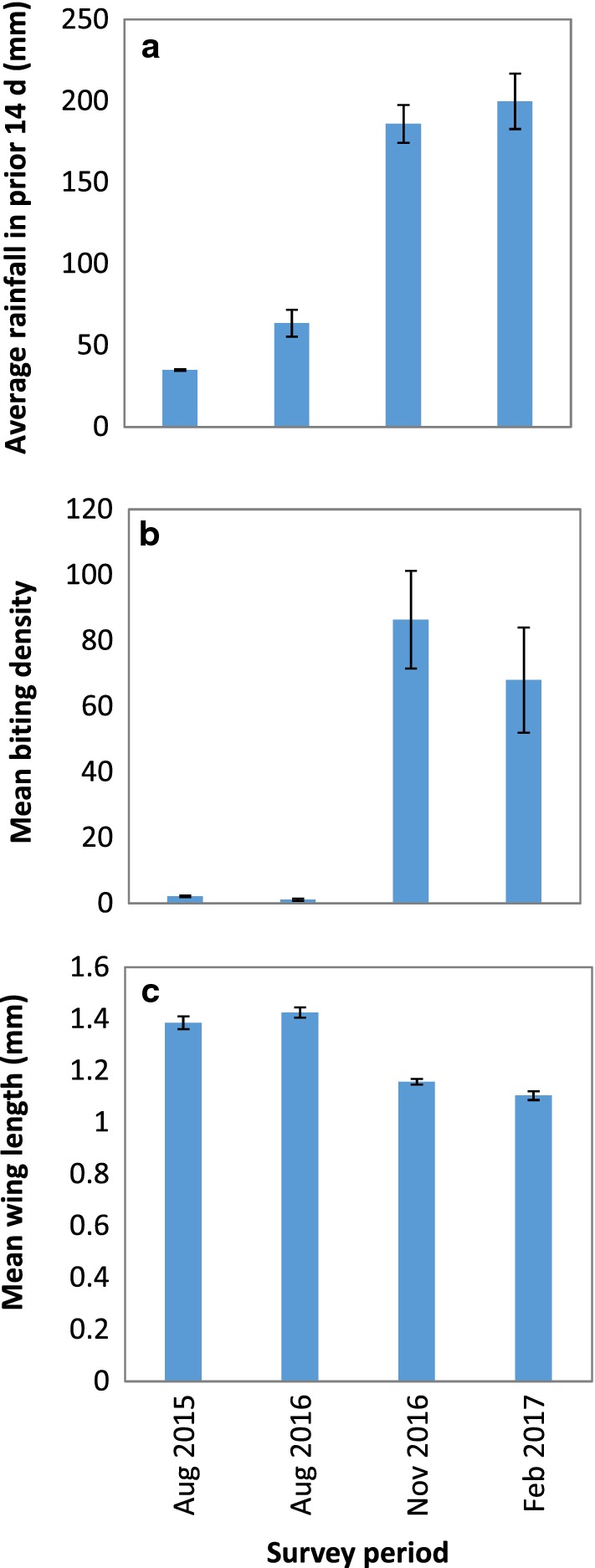
Comparison of independent sample periods in Jack Harbour with **a** rainfall, **b** *Anopheles farauti* densities and **c** mean wing lengths for. Each bar represents the average value (± se)

The density of *An. farauti* varied significantly in Jack Harbour by sampling period (*β* = 0.387, *SE* = 0.039, *P* < 0.0001; Fig. 7), and was thereby incorporated into the sequential GLMMs as a random factor. The density of host seeking *An. farauti* was positively associated with rainfall in the 14-day window prior to mosquito collections (*β* = 0.0243, *SE* = 0.0036, *P* < 0.0001, Fig. 7), with larger *An. farauti* populations being negatively associated with the size of the individual *An. farauti* (*β* = − 0.0002, *SE* = 0.0046, *P* = 0.007, Fig. 7).

A spatial analysis of the distribution of the wing size of *An. farauti* in Jack Harbour was unable to identify any biologically meaningful patterns, indicating that there is one interspersed population within the isolated village. Although the spatial analysis did identify significant clusters with larger mosquitoes (see Additional file 1), each cluster contained only one or two sampling locations equally dispersed across the village consistent with *An. farauti* being a single population.

## Discussion

Malaria transmission efficacy is a function of multiple vector parameters including size of the biting anopheline populations, survivorship, human blood feeding frequency and susceptibility to infection [34, 35], with the population dynamics of most taxa being influenced by both environmental and density dependent processes [36, 37]. The size of the biting populations and survivorship are two dominant transmission parameters with the potential to be impacted by density dependent feedback. For mosquito species, the influence of density dependent feedback on population growth is often overlooked, as population growth is heavily influenced by environmental factors, such as temperature, water and resource availability (exogenous processes) [10–12].

Considering that the size of adult mosquitoes is governed by competition during the immature aquatic stages, understanding the population dynamics of mosquitoes in larval habitats is important. Many anophelines larvae are found in large habitats (swamps, lagoons, ponds) [27], for which the relationship between larval density and adult fitness has only been recently demonstrated [13, 38]. In the Solomon Islands, *An. farauti* are often associated with large fresh or brackish water swamps (e.g., Jack Harbour and Haleta villages). These habitats are believed to produce most of the adult *An. farauti* despite the low densities of larvae found (unpublished data).

Here, the finding that the mean *An. farauti* body size was negatively associated with higher densities suggests a density dependent feedback occurring during the larval stages. The smallest *An. farauti* were found in Jack Harbour, which is dominated by a large swamp as the primary larval habitat. This habitat consistently had low densities of larvae (unpublished data). It is hypothesised that density dependent effects are outcomes affected by a combination of both the density of the larval population and the ability of the environment to support the population. Our observations suggest that this swamp habitat may possibly be quite nutrient poor and thus density dependent effects may be exerted as small *An. farauti* adults from low larval densities. Following rainfall, *An. farauti* populations increased in number and these more abundant mosquitoes were smaller still in size; this observation is also consistent with interacting environmental and density dependent influences on *An. farauti* populations (the relationship between rainfall and anopheline population size is well documented for a range of anopheline species [39–41]).

A previous study in these same villages established an association between the human biting rate of *An. farauti* and a malaria transmission focus in Jack Harbour [29]. Significant differences in mean *An. farauti* sizes among the villages within and outside this high malaria transmission focus were observed with the smallest *An. farauti* found in the malaria focus. However, identical estimates of *An. farauti* survivorship (by parous rates) were found in the focus village (Jack Harbour) and a village outside the focus (New Mala) [29], suggesting that any impact on survivorship associated with adult size was not of a magnitude that could be measured. Furthermore, the peak transmission period in the high malaria focus village corresponded with the period of highest abundance but smallest sized *An. farauti* [13]. This suggests that density dependence effects were insufficient to limit malaria transmission in the Solomon Islands, and that the density of *An. farauti* adult population is the strongest determinant of malaria transmission rates. This is not consistent with the dogma of smaller mosquitoes being less fit and therefore not as likely to survive long enough to transmit malaria [10, 15, 28]. Similarly, previous research with *Anopheles gambiae* in Tanzania, has observed that the success of host seeking females is not linked to population densities [11]. The observation in this study (that a population of smaller mosquitoes are not always less fit) is based on data from only two villages (in which a sufficient sample of mosquitoes could be collected to estimate parity) and would need confirming by additional observations. Any fitness loss associated with smaller mosquitoes appears to be outweighed by greater impact on transmission resulting from the greater numbers of mosquitoes present during the high transmission season.

## Conclusions

The findings here support a number of hypotheses. Firstly, *An. farauti* populations are directly associated with rainfall and inversely associated with the body size of individual mosquitoes. Secondly, density dependence impacts are likely occurring in a variety of habitats including large habitats. These impacts are expressed as variations in adult *An. farauti* size. The smallest *An. farauti* occurred during the peak transmission season suggesting that small mosquitoes are capable of adequately surviving long enough to transmit malaria. These results minimise concerns about whether density dependence might produce fitter vectors and suggests that decisions on whether to integrate larval control with ITN or IRS strategies for malaria vector control should be based predominantly on the capacity of national vector borne diseases control programs to effectively treat larval habitats.

## Additional file

**Additional file 1.** Spatial clusters of locations where larger *An. farauti* were captured within Jack Harbour as detected with SatScan.

## Data Availability

The dataset supporting the conclusions of this article are available in the JCU Tropical Data Hub repository at: 10.25903/5caedbc8a62cb.

## Abbreviations

API: annual parasite incidence
HLC: human landing catch
IRS: indoor residual spraying
LLIN: long lasting insecticidal net
LSM: larval source management
SIMHMS: Solomon Islands Ministry of Health and Medical Services
WHO: World Health Organization
