# Supplementary material for: Smallest Anopheles farauti occur during the peak transmission season in the Solomon Islands
Source: Malar J. 2019 Jun 24;18:208. doi: 10.1186/s12936-019-2847-2 (PMC6591980; doi:10.1186/s12936-019-2847-2)
Supplement: Supplementary file 1 — Additional file 1. Spatial clusters of locations where larger An. farauti were captured within Jack Harbour as detected with SatScan. [file 12936_2019_2847_MOESM1_ESM.docx]

**Additional files**

**Additional file 1:** Spatial clusters of locations where larger *An. farauti* were captured within Jack Harbour as detected with SatScan

| **LocationID** | **Number of Cases** | **Mean Inside** | **Mean Outside** | **Variance** | **Standard Deviation** | **Log Likelihood Ratio** | **P-Value** |
| --- | --- | --- | --- | --- | --- | --- | --- |
| 9 | 81 | 1.21 | 1.16 | 0.0011 | 0.034 | 64.425 | 0.001 |
| 10 | 147 | 1.20 | 1.16 | 0.0012 | 0.034 | 53.316 | 0.001 |
| 4,5 | 155 | 1.19 | 1.16 | 0.0013 | 0.036 | 26.264 | 0.001 |
| 3 | 34 | 1.20 | 1.17 | 0.0013 | 0.036 | 13.498 | 0.001 |
